# COVID-19 pandemic, healthcare providers’ contamination and death: an international view

**DOI:** 10.1186/s13054-020-02938-y

**Published:** 2020-05-08

**Authors:** Hossein Hassanian-Moghaddam, Nasim Zamani, Ali-Asghar Kolahi

**Affiliations:** 1 Social Determinants of Health Research Center, Shahid Beheshti University of Medical Sciences, Tehran, 1546817613 Iran; 2 Department of Clinical Toxicology, Loghman Hakim Hospital, School of Medicine,, Shahid Beheshti University of Medical Sciences, Tehran, Iran

Dear Editor,

There is a growing number of healthcare providers (HCPs) who have died as a result of the COVID-19 pandemic. In the past, the 2003 outbreak of severe acute respiratory syndrome (SARS) transmitted the virus to HCPs, who accounted for a fifth of all infected cases globally. Risk factors included poor institutional infection control measures and poor compliance with the use of personal protection equipment (PPE), among others [1]. Although there are novel guidelines for the prevention, quarantine, epidemiology, and treatment of COVID-19, little is known about its rates of occupational transmission to HCPs. At the time of writing, the total number of coronavirus cases in the world has already surpassed two million [2]. In the absence of official data, our preliminary search of media sources shows that 486 HCPs with an average age (SD) of 59 (14) years have died from COVID-19 ([3], Table 1), equivalent to an estimated death toll of 0.36% of HCPs relative to all registered COVID-19 deaths in the world. Due to limited data and possible underreporting, the true scale of deaths among HCPs remains unknown.

**Table 1:** Data on COVID-19 deaths in most affected countries with more than 10 HCP mortality

Country	Total mortality	Mortality of HCP	HCP’s death toll (%)	Date of report
Italy	21,645	120	0.55	April 7, 2020*
Iran	4777	95	1.99	April 16, 2020
USA	26,047	66	0.25	April 15, 2020**
UK	12,107	41	0.34	April 14, 2020^⊗^
Spain	14,792	26	0.18	April 08, 2020^Ψ^
Philippines	335	24	7.16	April 15, 2020**
China	1665	22	1.32	February 15, 2020^∅^
Indonesia	459	17	3.70	April 15, 2020**
Brazil	1532	17	1.11	April 15, 2020**
France	13,197	15	0.11	April 10, 2020^⊕^

*https://edition.cnn.com/world/live-news/coronavirus-pandemic-04-07-20/h_b7583ec9fa05d053f6faca9b164817eb

**https://www.medscape.com/viewarticle/927976#vp_1

^⊗^https://www.telegraph.co.uk/news/0/nhs-workers-died-coronavirus-frontline-victims/

^Ψ^https://cadenaser.com/ser/2020/04/08/sociedad/1586325379_363621.html

^∅^www.businessinsider.com/why-coronavirus-killed-young-chinese-doctors-2020-2

^⊕^https://www.20minutes.fr/sante/2758699-20200410-coronavirus-parmi-victimes-covid-19-france-professionnels-sante-paient-lourd-tribu

As depicted in the table, there is a huge gap between the highest reported death toll among HCPs in the Philippines (7.16%) and the lowest in France (0.11%). Notably, a death toll below 1% is only listed for high-income countries. Possible reasons for the variety in mortality rates between countries include the levels of preparedness, COVID-19 testing, and availability of PPE, as well as different protocols around release from the workplace to be quarantined.

In many countries, confirmation of disease is based on clinical characteristics and CT findings and not on possible exposure and laboratory confirmation. Many COVID-19 carriers therefore remain undetected, while HCPs on the frontlines face a high risk of contracting the disease. This includes HCPs working without sufficient PPE in ordinary departments where they face risk of exposure to unsuspected patients as well as HCPs in departments dedicated to confirmed COVID-19 cases.

The US Centers for Disease Control and Prevention have released useful information on interim infection prevention and control recommendations for COVID-19 patients in healthcare facilities, but implementation may not be feasible in low-resource settings with high patient volumes [4]. Therefore, we must stay ahead of the game in preventing the transmission of COVID-19 to HCPs; otherwise, we will quickly run out of human resources even if we have enough supplies. Let us save our rescuers!

## Data Availability

The data is all presented in the text.
